# Diffuse Large B‐Cell Lymphoma Presenting in a Background of Rosai–Dorfman Disease

**DOI:** 10.1155/crh/6560267

**Published:** 2026-05-17

**Authors:** Chao Wu, Anders Meyer, Aung M. Tun

**Affiliations:** ^1^ Department of Internal Medicine - Residency, The University of Kansas Medical Center, Kansas City, Kansas, USA, kumc.edu; ^2^ Department of Pathology, University of Kansas Medical Center, Kansas City, Kansas, USA, kumc.edu; ^3^ Division of Hematologic Malignancies and Cellular Therapeutics, The University of Kansas Medical Center, Kansas City, Kansas, USA, kumc.edu

## Abstract

Rosai–Dorfman disease (RDD) can present with lymphadenopathy. However, it is important to note that RDD can also coexist with hematologic malignancies, including non‐Hodgkin lymphomas. We present a case of an older male patient with generalized lymphadenopathy who was first diagnosed with RDD. High‐dose steroid therapy was rendered without a satisfactory response. Thus, the tissue specimens were re‐examined, and the patient was found to have diffuse large B‐cell lymphoma (DLBCL) in the background of RDD. The laboratory tests are notable for a slightly elevated serum lactate dehydrogenase (LDH) level. A positron emission tomography‐computed tomography scan revealed generalized hypermetabolic lymphadenopathy above and below the diaphragm. A bone marrow biopsy showed normocellular marrow without evidence of DLBCL. The international prognostic index score was 3 (age > 60 years, elevated serum LDH level, and Stage III disease), indicating a high‐intermediate risk disease. The patient was treated with six cycles of golcadomide in combination with R‐Pola‐CHP (rituximab, polatuzumab vedotin, cyclophosphamide, doxorubicin, and prednisone) for the underlying DLBCL, with resolution of both DLBCL and RDD. The disease remained in remission at the follow‐up visit 9 months after completing the therapy. This case highlights the importance of recognizing underlying DLBCL in patients with RDD for optimal patient outcomes.

**Trial Registration:** ClinicalTrials.gov identifier: NCT04884035

## 1. Introduction

Rosai–Dorfman disease (RDD) is a rare histiocytic malignancy generally affecting children but also adults of any age with a slight male predominance [1]. Patients present with significant lymphadenopathy and extranodal site involvement. Rarely, RDD is also observed in patients with Hodgkin and non‐Hodgkin lymphomas, with approximately 34 cases having been reported in the literature [1]. RDD coexisting with diffuse large B‐cell lymphoma (DLBCL) is exceptionally rare, with only 4 cases reported in the literature to date [1–6]. It is prudent to recognize and treat such cases in a timely manner, as a delay in the management of DLBCL can result in devastating patient outcomes.

## 2. Case Presentation

A male in his 70s initially presented with a progressive, painful lump on the left side of the neck along with night sweats. He denied significant weight loss, loss of appetite, shortness of breath, or mucocutaneous lesions. The physical examination was notable for the left tonsillar, cervical, supraclavicular, and axillary lymphadenopathy, as well as the right inguinal adenopathy. The laboratory tests did not show cytopenia. The serum lactate dehydrogenase (LDH) level was slightly elevated. Follicle‐stimulating hormone, luteinizing hormone, testosterone, prolactin, and thyroid‐stimulating hormone levels were within the normal range. The erythrocyte sedimentation rate was 50 mm/hour. The bone marrow biopsy showed normocellular marrow, 40%, with appropriate trilineage haematopoiesis. The magnetic resonance imaging of the brain showed a 2 mm hypo‐enhanced lesion in the posterior pituitary gland. A positron emission tomography‐computed tomography (PET/CT) revealed diffuse, generalized hypermetabolic lymphadenopathy above and below the diaphragm (Figure 1(a)).

FIGURE 1(a) Baseline PET/CT scan showing generalized lymphadenopathy and (b) PET/CT scan showing complete resolution of lymphadenopathy post treatment.

**Figure:**
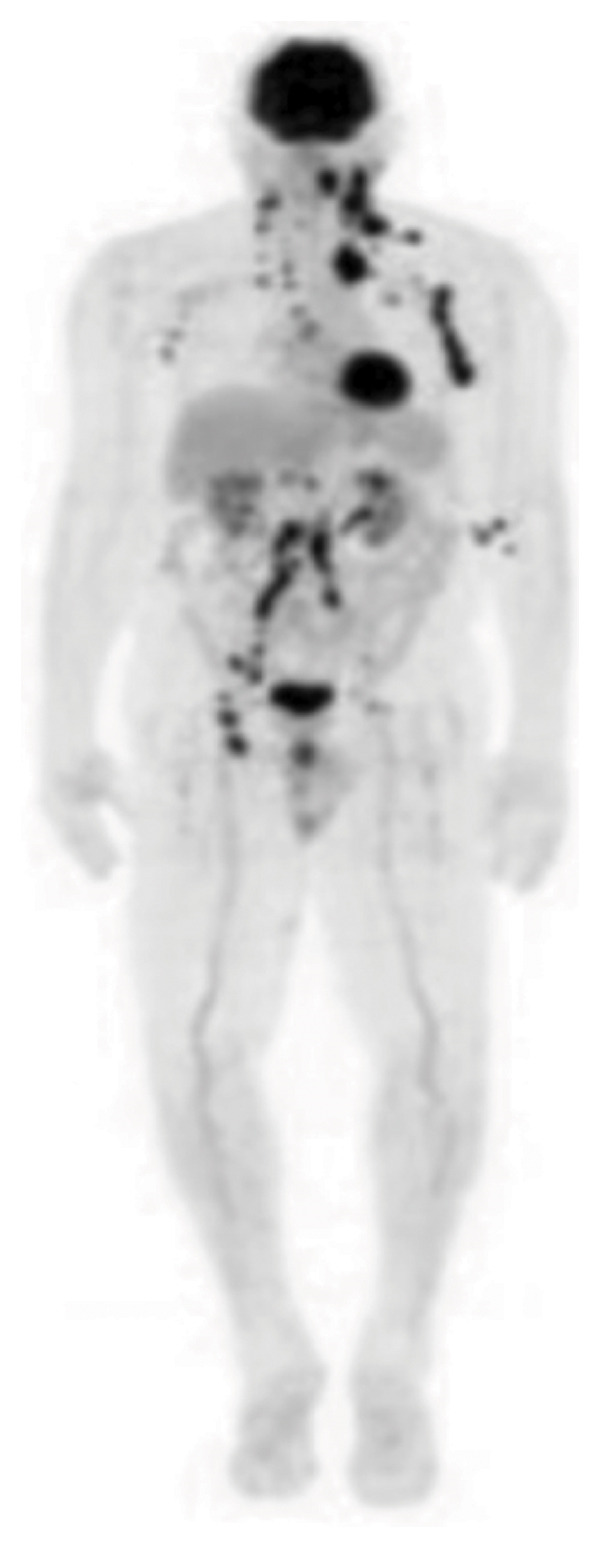
(a)

**Figure:**
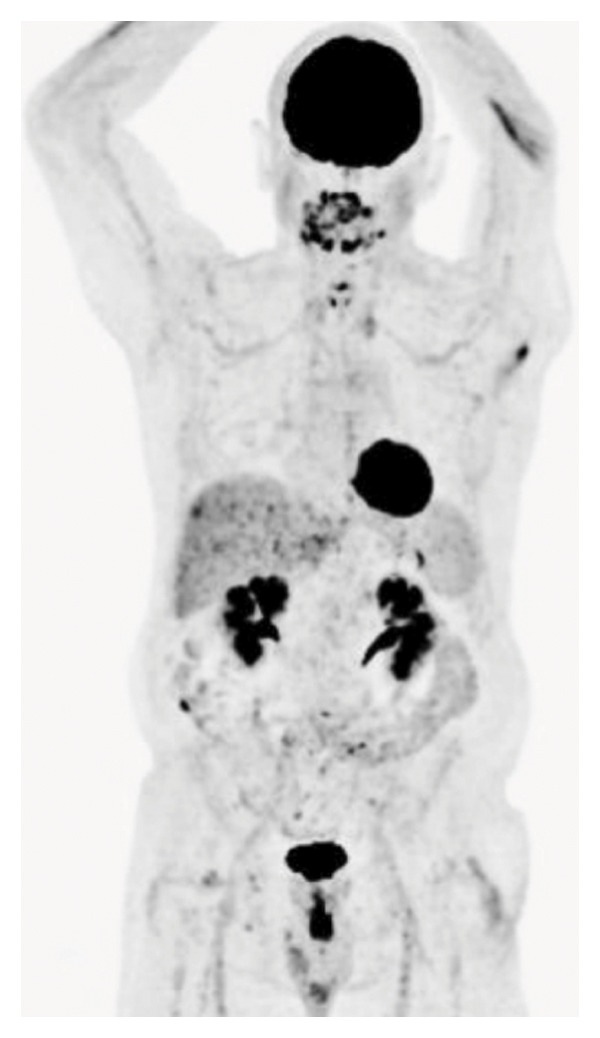
(b)

**FIGURE 2:**
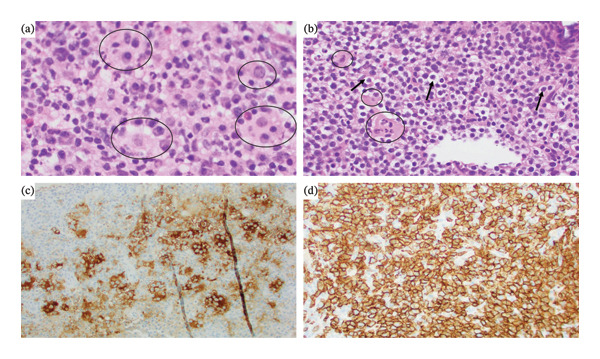
(a) Large, eosinophilic histiocytes admixed with and containing neutrophils, plasma cells, and rare eosinophils; (b) focus showing decreased large eosinophilic histiocytes and increased background monomorphic lymphocytes; (c) S100 immunohistochemistry staining the large histiocytes and demonstrating emperipolesis; and (d) CD20 immunohistochemistry showing dense and diffuse staining of the background lymphocytes. Black circles highlight large histiocytes consistent with RDD. Black arrows point to neoplastic lymphocytes consistent with DLBCL. Note: There are many more histiocytes and lymphocytes present that can be indicated.

Serum protein electrophoresis showed hypoalbuminemia and polyclonal gammopathy consistent with chronic inflammation, while immunoglobulin levels revealed slight elevation in IgG and IgA and normal IgM. C‐reactive protein level was elevated at 3.09 mg/dL. The core needle biopsy and histopathology evaluation from the right inguinal lymph node showed large eosinophilic histiocytes with cellular emperipolesis that were positive for S100 and CD68 but negative for SOX10 and CD1a (Figures 2(a), 2(b), and 2(c)), consistent with RDD. Subsequent workup with next‐generation sequencing was negative for mutations in the BRAF and MAP kinase pathways and also negative for the FAS gene. As high‐dose steroid therapy failed to achieve a satisfactory response, the histopathology specimens were re‐examined, revealing neoplastic lymphocytes positive for CD20 (Figure 2(d)), BCL‐6, CD10, BCL‐2, and c‐MYC but negative for IgG4 cells with a Ki‐67 proliferation index of 85%. These findings were consistent with DLBCL, a germinal center B‐cell (GCB) subtype.

After establishing a diagnosis of Stage IIIA DLBCL in the background of RDD, the patient was enrolled into a clinical study and treated with six cycles of golcadomide, a novel immunomodulator drug of the E3 ubiquitin ligase complex, in combination with R‐Pola‐CHP (rituximab, polatuzumab vedotin, cyclophosphamide, doxorubicin, and prednisone) [7]. A complete metabolic response was achieved on the end of the treatment PET/CT scan (Figure 1(b)). There was no evidence at lymphadenopathy or organomegaly on the follow‐up CT scans done 9 months later.

## 3. Discussion

RDD is a rare histiocytic malignancy that primarily occurs in children but is also seen in adults with a slight male predominance [1]. The disease can present as localized or generalized lymphadenopathy and/or involve extranodal sites, with the skin, soft tissue, upper respiratory tract, multifocal bone, and orbital tissue being common sites [8–10]. RDD has been recognized since the 1960s, and the disease itself continues to be quite rare [11, 12]. The histopathologic findings of large histiocytic cells with round hyperchromatic nuclei, distinctive centralized nucleoli, and an abundant pale cytoplasm are required for a diagnosis of RDD [8, 10]. Emperipolesis, a phenomenon of nondestructive leukocyte engulfment in the cytoplasm, is frequently observed, though not necessarily a disease‐specific feature. The pathologic hallmarks include the presence of CD68‐positive, CD163‐positive, CD14‐positive, S100‐positive, and CD1a‐negative histiocytic cells [1, 8, 10]. Mutually exclusive KRAS and MAP2K1 mutations can be seen in one‐third of patients with RDD [13]. Of note, a subset of patients with RDD can have polyclonal hypergammaglobulinemia and the presence of IgG4‐positive plasma cells within lymph nodes and other tissues along with elevations in serum IgG4 levels [14].

Rarely, RDD can be observed in patients with Hodgkin and non‐Hodgkin lymphomas along with other hematologic malignancies [1, 15]. Approximately 34 cases of RDD co‐occurring with lymphomas have been reported [1]. The pathogenic connection between RDD and lymphoma is not well understood, but it is hypothesized that cytokines in the lymphoma microenvironment induce the accumulation of histiocytic cells characteristic of RDD [15]. Of those aforementioned cases, approximately 4 previous cases have been documented to have had some level of co‐existence with DLBCL [1–6]. It is also worth mentioning that there are individual cases of DLBCL being detected in patients with a documented history of RDD, though separated temporally. Individual cases such as these are hypothesized to be lymphoma transformed from RDD, though the specifics on how the events are correlated are not fully clear [3]. This particular presentation makes at least the fifth case of co‐existence. Previously, the phenomenon of RDD proper was often considered clinically benign. However, it is now considered a clonal myeloid neoplasm, with 35%–40% of RDD demonstrating a mutation in the MAPK pathway which can be targetable with MEK inhibitors [16]. Regardless, when coexisting with a known lymphoma, the cornerstone of management still pertinently includes treating the underlying lymphoma. [15].

For this patient’s disease, the international prognostic index score was 3 (age > 60 years, elevated serum LDH level, and Stage III disease), indicating a high‐intermediate risk disease. In such patients, the chemoimmunotherapy regimen R‐Pola‐CHP is shown to be superior to R‐CHOP (rituximab, cyclophosphamide, doxorubicin, vincristine, and prednisone) based on the Phase III, randomized POLARIX trial, though the subgroup analysis did not show clear improvement in patients with the GCB subtype. [17].

This case highlights the importance of recognizing underlying lymphoma, particularly DLBCL, in patients with lymphadenopathy and histopathology evidence of RDD. A high degree of suspicion is needed for a timely diagnosis and management of this rare presentation.

## Author Contributions

All authors are involved in the preparation of the manuscript.

Aung M. Tun had full access to all of the data in this study and takes complete responsibility for the integrity of the data and the accuracy of the data analysis.

## Funding

No funding was received for this manuscript.

## Disclosure

All authors have read and approved the final version of the manuscript.

## Conflicts of Interest

The authors declare no conflicts of interest.

## Data Availability

The data that support the findings of this study are available on request from the corresponding author. The data are not publicly available due to privacy or ethical restrictions.

## References

[1] Bruce-Brand C. , Schneider J. , and Schubert P. , Rosai-Dorfman Disease: An Overview, Journal of Clinical Pathology. (2020) 73, no. 11, 697–705, 10.1136/jclinpath-2020-206733. 32591351

[2] Moore J. , Zhao X. , and Nelson E. , Concomitant Sinus Histiocytosis With Massive Lymphadenopathy (Rosai-Dorfman Disease) and Diffuse Large B-Cell Lymphoma: A Case Report, Journal of Medical Case Reports. (2008) 2, no. 1, 10.1186/1752-1947-2-70, 2-s2.0-45149129105. PMC2270859 18321383

[3] Kaçka D. , Ukimeraj A. , Çavolli V. , and Hoda K. , ABCL-028 Transformed Non-Hodgkin Lymphoma: A Case Originating from Rosai-Dorfman Disease-Case Presentation, Clinical Lymphoma, Myeloma and Leukemia. (2024) 24, no. 2024, 10.1016/S2152-2650(24)01479-4.

[4] Shoda H. , Oka T. , Inoue M. et al., Sinus Histiocytosis With Massive Lymphadenopathy Associated With Malignant Lymphoma, Internal Medicine. (2004) 43, no. 8, 741–745, 10.2169/internalmedicine.43.741, 2-s2.0-4644293848. 15468978

[5] Krzemieniecki K. , Pawlicki M. , Marganska K. , and Parczewska J. , The Rosai-Dorfman Syndrome in a 17-Year-Old Woman With Transformation Into High-Grade Lymphoma. A Rare Case Presentation, Annals of Oncology. (1996) 7, no. 9, 10.1093/oxfordjournals.annonc.a010804, 2-s2.0-0030468311. 9006752

[6] Melikian A. , Kovrigina A. , Giliazitdinova E. , and Gitis M. , A Case of Sinus Histiocytosis With Massive Lymphadenopathy (Rosai-Dorfman Disease) in a Patient with Diffuse Large B-Cell Lymphoma and Chronic Hepatitis B Virus Infection, Terapevticheskii Arkhiv. (2012) 84, no. 11, 66–70, https://medbiosci.ru/0040-3660/article/view/31151. 23252252

[7] Tun A. and Ansell S. , Immunotherapy in Hodgkin and Non-Hodgkin Lymphoma: Innate, Adaptive and Targeted Immunological Strategies, Cancer Treatment Reviews. (2020) 88, 10.1016/j.ctrv.2020.102042. 32521386

[8] Abla O. , Jacobsen E. , Picarsic J. et al., Consensus Recommendations for the Diagnosis and Clinical Management of Rosai-Dorfman-Destombes Disease, Blood. (2018) 131, no. 26, 2877–2890, 10.1182/blood-2018-03-839753, 2-s2.0-85049122426. 29720485 PMC6024636

[9] Goyal G. , Ravindran A. , Young J. et al., Clinicopathological Features, Treatment Approaches, and Outcomes in Rosai-Dorfman Disease, Haematologica. (2020) 105, no. 2, 348–357, 10.3324/haematol.2019.219626. 31004029 PMC7012468

[10] Goyal G. , Young J. , Koster M. et al., The Mayo Clinic Histiocytosis Working Group Consensus Statement for the Diagnosis and Evaluation of Adult Patients With Histiocytic Neoplasms: Erdheim-Chester Disease, Langerhans Cell Histiocytosis, and Rosai-Dorfman Disease, Mayo Clinic Proceedings. (2019) 94, no. 10, 2054–2071, 10.1016/j.mayocp.2019.02.023, 2-s2.0-85071315667. 31472931

[11] Henter J. , Tondini C. , and Pritchard J. , Histiocyte Disorders, Critical Reviews in Oncology. (2004) 50, no. 2, 157–174, 10.1016/j.critrevonc.2004.01.002, 2-s2.0-2442480880. 15157664

[12] Foucar E. , Rosai J. , and Dorfman R. , Sinus Histiocytosis With Massive Lymphadenopathy (Rosai-Dorfman Disease): Review of the Entity, Seminars in Diagnostic Pathology. (1990) 7, no. 1, 19–73, https://pubmed.ncbi.nlm.nih.gov/2180012/. 2180012

[13] Garces S. , Medeiros L. , Patel K. et al., Mutually Exclusive Recurrent KRAS and MAP2K1 Mutations in Rosai–Dorfman Disease, Modern Pathology. (2017) 30, no. 10, 1367–1377, 10.1038/modpathol.2017.55, 2-s2.0-85030623937. 28664935 PMC5837474

[14] Chen L. , Slack G. , and Carruthers M. , IgG4-Related Disease and Rosai-Dorfman-Destombes Disease, Lancet. (2021) 398, no. 10307, 1213–1214, 10.1016/S0140-6736(21)01812-2. 34600619

[15] Garces S. , Yin C. , Patel K. et al., Focal Rosai–Dorfman Disease Coexisting With Lymphoma in the Same Anatomic Site: A Localized Histiocytic Proliferation Associated With MAPK/ERK Pathway Activation, Modern Pathology. (2019) 32, no. 1, 16–26, 10.1038/s41379-018-0152-1, 2-s2.0-85055051612. 30323237

[16] Abeykoon J. , Rech K. , Young J. et al., Outcomes After Treatment With Cobimetinib in Patients With Rosai-Dorfman Disease Based on KRAS and MEK Alteration Status, JAMA Oncology. (2022) 8, no. 12, 1816–1820, 10.1001/jamaoncol.2022.4432. 36201194 PMC9539729

[17] Tilly H. , Morschhauser F. , Sehn L. et al., Polatuzumab Vedotin in Previously Untreated Diffuse Large B-Cell Lymphoma, New England Journal of Medicine. (2022) 386, no. 4, 351–363, 10.1056/NEJMoa2115304. 34904799 PMC11702892

